# Hepatic Olfr734 Deficiency Worsens Hepatic Glucose Metabolism and Induces MASLD in Mice

**DOI:** 10.3390/nu17152426

**Published:** 2025-07-25

**Authors:** Eva Prida, Diego Muñoz-Moreno, Eva Novoa, Tamara Parracho, Laura Diaz-Garzón Dopico, Raquel Perez-Lois, Miguel Bascoy-Otero, Ana Senra, Sergio Romero-Rodriguez, Beatriz Brea-García, Jaime Dobarro, Adrián Fernández Marcos, Javier Baltar, Fernando Santos, Amaia Rodríguez, Gema Frühbeck, Ruben Nogueiras, Luisa María Seoane, Mar Quiñones, Omar Al-Massadi

**Affiliations:** 1 Translational Endocrinology Group, Endocrinology Section, Instituto de Investigación Sanitaria de Santiago de Compostela, Complexo Hospitalario Universitario de Santiago (IDIS/CHUS), Travesía da Choupana s/n, 15706 Santiago de Compostela, Spain; eva.prida@rai.usc.es (E.P.); lauradgd@gmail.com (L.D.-G.D.); beabreag@hotmail.com (B.B.-G.); dobarro89@gmail.com (J.D.); adrianangelfm@gmail.com (A.F.M.); 2 CIBER Fisiopatología de la Obesidad y Nutrición (CIBERobn), Av. Monforte de Lemos 3-5, 28029 Madrid, Spain; diego.munoz0@rai.usc.es (D.M.-M.); evam.novoa@usc.es (E.N.); tamara.parracho.martinez@usc.es (T.P.); loisperezraquel@gmail.com (R.P.-L.); miguel.bascoy@rai.usc.es (M.B.-O.); sergio.romero.rodriguez@rai.usc.gal (S.R.-R.); arodmur@unav.es (A.R.); gfruhbeck@unav.es (G.F.); ruben.nogueiras@usc.es (R.N.); 3 Grupo Fisiopatología Endocrina, Área de Endocrinología, Instituto de Investigación Sanitaria de Santiago de Compostela, Travesía da Choupana s/n, 15706 Santiago de Compostela, Spain; 4 Department of Physiology, Center for Research in Molecular Medicine and Chronic Diseases, Instituto de Investigación Sanitaria de Santiago de Compostela, University of Santiago de Compostela, 15782 Santiago de Compostela, Spain; ana.senra@usc.es; 5 Servicio de Cirugía General y del Aparato Digestivo, Complexo Hospitalario Universitario de Santiago/Servizo Galego de Saude, Rua R Baltar s/n, 15706 Santiago de Compostela, Spain; javier.baltar.boileve@sergas.es (J.B.); ffsantosbenito@gmail.com (F.S.); 6 Metabolic Research Laboratory, Cancer Center Clínica Universidad de Navarra (CCUN), 31008 Pamplona, Spain; 7 Obesity and Adipobiology Group, Instituto de Investigación Sanitaria de Navarra (IdiSNA), 31008 Pamplona, Spain; 8 Department of Endocrinology & Nutrition, Cancer Center Clínica Universidad de Navarra (CCUN), Avda. Pío XII, 36, 31008 Pamplona, Spain; 9 Galician Agency of Innovation (GAIN), Xunta de Galicia, 15702 Santiago de Compostela, Spain

**Keywords:** asprosin, Olfr734, MASLD, hepatic glucose production

## Abstract

**Background/Objectives:** Asprosin is the endogenous ligand of the olfactory Olfr734 receptor linked to MASLD and glucose metabolism. Despite the involvement of asprosin in these processes, little has been published on the specific role of Olfr734 in liver function. The aim of this work is therefore to study the specific role of the olfactory Olfr734 receptor in MASLD and glucose metabolism. **Methods:** To achieve this objective, we performed a genetic inhibition specifically to inhibit Olfr734 in the livers of male mice. We then studied the progression of MASLD in DIO mice. In addition, we studied the glucose metabolism in hypoglycemia states and postprandial glucose production in standard diet-fed mice. Finally, analyses of liver biopsies from patients with obesity and with or without T2DM were conducted. **Results:** We found that hepatic Olfr734 levels vary according to changes in nutritional status and its knockdown effect in the liver is to increase the hepatic lipid content in DIO mice. Our results also showed that OLFR734 expression is involved in the adaptive response in terms of glucose production to nutrient availability. Finally, the hepatic human Olfr734 ortholog named OR4M1 has been observed to be at significantly higher levels in male patients with T2DM. **Conclusions:** This study increases understanding of the mechanisms by which the modulation of Olfr734 expression affects liver function.

## 1. Introduction

Metabolic dysfunction-associated steatotic liver disease (MASLD) is a metabolic disorder characterized by the convergence of hepatic steatosis and at least one cardiometabolic risk factor, which can progress from simple steatosis to steatohepatitis, fibrosis, cirrhosis, and hepatocellular carcinoma [1]. MASLD is among the most prevalent liver diseases associated with obesity, type 2 diabetes mellitus (T2DM), and metabolic syndrome [2,3,4]. Current data indicate that the prevalence of MASLD in the general population is approximately 30% [5], and ranges from 42.6% to 69.5% in patients with obesity or T2DM [2,3,6,7]. The prevalence of liver diseases is rising, primarily due to increasing obesity rates worldwide. Despite recent advances made by the research community, the pharmacological treatments currently available for treating liver diseases remain limited [8,9,10]. There is therefore an urgent need for new treatments in order for health outcomes to be improved and thus reduce the burden associated with MASLD. Identifying new molecular targets is therefore imperative.

Asprosin is a hormone predominantly produced by white adipose tissue (WAT) and plays a crucial role in energy balance regulation [11]. The hormone is the C-terminal cleavage product of profibrillin, and, in humans, the absence of its gene results in extreme leanness [11]. Furthermore, it has been demonstrated that both short- and long-term changes in nutritional status, such as fasting or obesity, lead to elevated levels of circulating asprosin in rodents and humans [11]. Other studies corroborate this finding, indicating that circulating levels of asprosin are positively correlated with obesity in children and adolescents [12] and in adult patients with T2DM [13]. In preclinical studies, asprosin has been shown to increase food intake by enhancing olfactory performance and activating hypothalamic AgRP neurons [14]. Furthermore, asprosin acts in the liver to augment glucose production [15].

Since its identification in 2016, new metabolic functions have been attributed to asprosin. Beyond its established role in obesity and T2DM, its influence on hepatic diseases has attracted increasing interest in recent years. The initial association between asprosin and liver disease was reported in epidemiological studies, which demonstrated a correlation between asprosin expression and the severity of MASLD in patients [16]. Preclinical investigations have corroborated these observations in humans. Specifically, two separate studies have indicated that genetic suppression of asprosin in adipose tissue or the liver ameliorates MASLD symptoms in diet-induced obese (DIO) mice by altering lipid metabolism enzymes and reducing inflammation [17,18]. While these studies mainly concentrated on modulating the hormone itself, there has only been limited study of its receptor, the olfactory receptor Olfr734, as a potential therapeutic target. This focus may well be of benefit in certain contexts, such as in the study of liver diseases or diabetes, where the liver’s role is pivotal and the hepatic asprosin levels are reported to be significantly lower compared to in other organs [15,19]. In this study, we observed that the hepatic Olfr734 levels fluctuate in response to nutritional status changes, and its knockdown in the liver elevates the hepatic lipid content and induces glucose production in DIO mice. Furthermore, to assess the translational relevance of our findings, we demonstrated that the human hepatic Olfr734 ortholog, OR4M1, is present at significantly higher levels in male patients with obesity and T2DM compared to those with obesity and normoglycemia.

## 2. Materials and Methods

### 2.1. Mouse Models

Male C57BL/6J wild-type (WT) mice, sourced from the Centro de Biomedicina Experimental de Galicia (CEBEGA) at the University of Santiago de Compostela (USC), Spain, aged 6 to 8 weeks, were utilized in this study. The animals were maintained under Specific-Pathogen-Free conditions, with a controlled temperature of 23 °C and a 12 h light/dark cycle. They had unrestricted access to water and were fed either a standard diet (STD; 6% kcal fat, 18% kcal protein, Teklad Global, Inotiv, West Lafayette, IN, USA) or a high-fat diet (HFD; 60% kcal from fat; D12492, Research Diets, New Brunswick, NJ, USA). All animal care procedures conformed to the guidelines of the institutional animal care committee, with all protocols reviewed and approved by the Ethics Committee of the USC, in accordance with EU regulations on the use of experimental animals (Project ID 15012/2023/012). Environmental enrichment was incorporated into the animal enclosures to promote the expression of natural behaviors and enhance overall welfare. This enrichment included materials such as nesting substrates, shelters, and objects designed for exploration and interaction.

Body weight and food intake were monitored on a weekly basis following lentivirus injection, with all researchers being informed of the group assignments of the animals. Animals exhibiting signs of illness or injuries from cage fights were excluded from the study. No measures were implemented to control for confounding factors during the experiment. The sample size was determined using the 3R principle to ensure statistical validity and significance. Mice were randomized based on body weight, with two groups of animals of the same age and similar body weights being allocated to each group. The precise number of animals utilized in each experiment is specified in the corresponding figure legend. Animals were euthanized by decapitation, and tissues were promptly collected, rapidly frozen using dry ice, and stored at −80 °C until further analysis.

### 2.2. Effect of the Diet and Food Deprivation on Hepatic Olfr734 Levels

Diet: After weaning, 6-week-old male C57BL/6J mice were either fed an HFD or an STD for 12 weeks [20].

Fasting: 10-week-old male C57BL/6J mice were deprived of food (STD) for 24 h, while the control group was fed ad libitum [20,21,22].

To study the role of nutrient deprivation in DIO mice on the hepatic levels of Olfr734, 6-week-old male C57BL/6J mice were fed with HFD for 12 weeks and were subjected to 24 h of fasting.

To study the hepatic inhibition of Olfr734 on glucose production during fasting, male C57BL/6J standard diet-fed mice were subjected to a 24 h fast [23,24].

All animals had free access to tap water.

### 2.3. Tail Vein Injection of Lentiviral Expression Vectors

The viruses were administered via the tail vein to specifically target the liver. Mice were secured in a specialized restrainer designed for intravenous injections (Tailveiner TV-150, Bioseb, Vitrolles, France). Using a 27 G × 3/8” syringe, 100 μL of 2 shRNA lentiviral particles, packaged from pGFP-C-shlentivector (Origene Technologies GmbH, Herford, Germany) or control shRNA, both diluted in saline [21,22], was injected. The following shRNA target sequences were employed:

TL515502VA-TGAGATGTTCCTGCTGACAGTGATGGCTT with a titer of 2.2 × 10^7^ TU/mL.

TL515502VB–TTATTCTCACTGGTCTATCTCAGACTCGG with a titer of 2.6 × 10^7^ TU/mL.

TR30021V Lenti shRNA scramble control particles with a titer of 1 *×* 10^7^ TU/mL.

In the experiments concerning MASLD, the knockdown of Olfr734 was induced in WT mice fed an HFD diet for 15 weeks, with the lentivirus shOlfr734 injected 6 weeks prior to euthanasia. In the study examining the role of Olfr734 inhibition on hepatic glucose production, WT C57BL/6 mice were fed an STD for 12 weeks, and the lentivirus shOlfr734 was injected in the first week.

### 2.4. Calorie Restriction

We assessed the food intake of each mouse over a period of five days to ascertain their daily consumption. Subsequently, the average intake was calculated, and their consumption was restricted by 60% for four days, with only 40% of the calculated quantity provided daily at 6 p.m. Body weight and blood glucose levels were recorded daily at 5:30 p.m. prior to the provision of additional food during the four-day experimental period [21,22].

### 2.5. Effect of Glucose on Hepatic Olfr734 Levels

WT mice were allocated into two groups: (a) those fed an ad libitum STD, and (b) those subjected to a 24 h fasting period, followed by ad libitum feeding with sucrose cubes for 24 h to maintain normal blood glucose levels in the absence of other nutrients, as previously described [21,22].

### 2.6. Glucose, Insulin, Pyruvate, and Postprandial Glucose Tolerance Tests

To assess glucose tolerance (GTT), insulin tolerance (ITT), and pyruvate tolerance (PTT), the mice underwent specific tests. For GTT, mice were intraperitoneally administered D-glucose (2 g/kg) following an overnight fast, and blood glucose levels were measured at intervals of 0, 15, 30, 60, 90, and 120 min using a glucometer (Accu-Chek, Roche, Basel, Switzerland). For ITT, mice received a bolus injection of insulin (0.5 IU/kg) after a 6 h fast, and blood glucose levels were measured at the same intervals. For PTT, sodium pyruvate (1.25 g/kg) was intraperitoneally administered following an overnight fast. Blood glucose levels were measured at intervals of 0, 20, 40, 60, 80, and 120 min [20,21,22,24].

A postprandial glucose tolerance test was conducted to assess plasma glucose levels following food consumption. After an overnight fast, measurements of blood glucose levels and body weight were taken. Feeding was subsequently resumed, and food intake, blood glucose levels, and body weight were recorded at intervals of 30 min, 60 min, 120 min, and 240 min [21,22].

### 2.7. Liver Triglycerides

Liver samples (approximately 500 mg) were homogenized for 2 min in ice-cold chloroform–methanol (2:1, *v*/*v*). Triglycerides (TG) were extracted by agitating the homogenate at room temperature for 3 h. To facilitate phase separation, Milli-Q water was added, followed by centrifugation. The organic (bottom) layer was collected, dried using a SpeedVac, and re-dissolved in chloroform. TG content was quantified in duplicate using an enzymatic assay (1001310 Spinreact, Girona, Spain) after the evaporation of the organic solvent.

### 2.8. Plasma Measurements

To evaluate the lipid profiles and liver function of the mice, plasma triglycerides (TG: 1001310, Spinreact; Girona, Spain), total cholesterol (1001093, Spinreact Girona, Spain), non-esterified free fatty acids (91696, Fujifilm, Tokio, Japan), and transaminases (ALT: 41283, and AST: 41273 Spinreact; Girona, Spain) levels were measured. Blood samples were obtained by puncture of the tail vein and then centrifuged at 1500× *g* for 10 min to obtain plasma. Post-mortem glucose (1001191, Spinreact; Girona, Spain) levels were also assessed from plasma. Plasma levels of insulin were established using an insulin (Merck Mili-pore, ref. EZRMI, Taufkirchen, Germany) ELISA kit. Readings were collected according to the manufacturer’s instructions, and results were expressed in MMOL/L or U/ML for each measured parameter [20,21,22,24,25].

### 2.9. SYBR Green One-Step RT-qPCR

The extraction of mRNA commenced with the resuspension of the sample in 750 µL of TRIzol, followed by the addition of 150 µL of chloroform (VWR Chemicals, Mississauga, ON, Canada). The mixture was subjected to vortexing and subsequently centrifuged at 12,000× *g* for 15 min at 4 °C. The aqueous phase was then isolated, and an equivalent volume of 70% ethanol (VWR Chemicals; Mississauga, ON, Canada) was incorporated. The phases were combined and transferred to a column utilizing an RNA extraction kit (Omega Bio-Tek, Norcross, GA, USA). The column underwent centrifugation at 10,000× *g* at 21 °C for one minute, after which the liquid was discarded. Subsequently, 500 µL of Wash Buffer I (Omega Bio-Tek RNA extraction kit; Norcross, GA, USA) was added, followed by another centrifugation at 10,000× *g* at 21 °C for one minute. The liquid was discarded once more, and 500 µL of Wash Buffer II was introduced. This centrifugation step at 10,000× *g* at 21 °C for one minute was repeated twice. Following the final wash, the column was centrifuged at 14,000× *g* for 2 min at 21 °C. Finally, 40 µL of RNAse-free water was added, and the sample was centrifuged at 14,000× *g* for 2 min at 21 °C to elute the RNA [20,25,26,27]. The one-step RT-qPCR was performed in 10 µL reaction volumes containing 10 ng of RNA template. RNA concentrations were determined spectrophotometrically using a NanoDrop (Thermo Scientific NanoDrop, Waltham, MA, USA). Each reaction comprised 0.4 µL of 10 µM forward primer, 0.4 µL of 10 µM reverse primer, 0.4 µL of NZYRT mix, 5 µL of One-step NZYSpeedy qPCR Probe Master Mix (2×) (NZYTech, Lisbon, Portugal), and 1.8 µL of nuclease-free water [26].

The thermal cycling protocol commenced with an initial reverse transcription step at 50 °C for 15 min, followed by polymerase activation at 95 °C for 2.5 min. The cycling protocol consisted of 40 cycles at 95 °C for 5 s and 60 °C for 45 s, utilizing a 96-well thermal cycler (QuantStudio 3, Applied Biosystems, Thermo Fisher Scientific, Waltham, MA, USA). A melt curve analysis was conducted with the following stages: 15 s at 95 °C, 1 min at 60 °C, and 1 s at 95 °C [28]. Primers were designed using PRIMER-BLAST (https://www.ncbi.nlm.nih.gov/tools/primer-blast/) accessed on 1 January 2025. The list of primers used is provided in Table S1.

Liver extracts were homogenized using a TissueLyser II (Qiagen, Venlo, The Netherlands) in cold BLYS buffer (TRIS 1M, EGTA 0.2M, EDTA 0.2M, TRITON x-1000, orthovanadate 0.1M, sodium fluoride, sodium pyrophosphate, and saccharose: Sigma-Aldrich, St. Louis, MO, USA) with protease and phosphatase inhibitors (Sigma-Aldrich, St. Louis, MO, USA). The lysates were centrifuged (30 min, 12,000 rpm, 4 °C) and quantified using the DC Protein Assay (BioRad, Hércules, CA, USA), with readings taken by a spectrophotometer (BioTek, Winooski, VT, USA). Equal amounts (20 µg/well) of total protein lysates from the liver were subsequently subjected to SDS-PAGE, electrotransferred onto a PVDF membrane, and incubated with Olfr734 (MBS8566720, Mybiosource, San Diego, CA, USA), ANGPL4 (40-9800 Thermo Fisher Scientific; Waltham, MA, USA), FGF21 (Abcam ab64857; Cambridge, UK), GDF-15 (Abcam ab39999; Cambridge, UK), mTOR (Cell Signaling #2972; Danvers, MA, USA), and pmTOR (Cell Signaling #2971; Danvers, MA, USA) protein antibodies diluted to 1/1000 to confirm the knockdown. Protein levels were normalized relative to β-actin (MFCD00164531, Sigma-Aldrich, St. Louis, MO, USA) diluted to 1/10,000 or vinculin (sc-73614; Santa Cruz Biotechnology, Dallas, TX, USA) diluted 1/1000 for each sample. Membranes were treated with a 5% BSA blocking buffer, and protein detection was performed using horseradish peroxidase-conjugated secondary antibodies. Antigen–antibody interactions were detected using a chemiluminescence technique according to the manufacturer’s instructions (Pierce ECL Western Blotting Substrate, Thermo Scientific, Waltham, MA, USA). Visualization was conducted through manual X-ray film development or the ChemiDoc Imaging System. Western blot quantification was carried out using ImageJ software (RRID: SCR_003070).

### 2.10. Histological Procedures

Hematoxylin and Eosin Staining: Liver samples were fixed in 4% formaldehyde for 24 h, followed by dehydration and paraffin embedding. Sections of 4 µm thickness were prepared using a microtome and stained with a standard hematoxylin and eosin alcoholic procedure as per the manufacturer’s instructions (BioOptica, Milan, Italy). Subsequently, sections were rinsed with distilled water, dried at 37 °C for 30 min, and mounted using a permanent (non-alcohol, non-xylene-based) mounting medium [20,25,26].

Oil Red O Staining: Frozen liver samples were sectioned into 10 µm slices using a cryostat and stained with filtered Oil Red O for 20 min (Sigma O-0625; St. Louis, MO, USA). Following washing in distilled water, sections were counterstained with Harris’s hematoxylin for 5 min (Bio-optica 05-06004/L; Milan, Italy) and mounted in an aqueous medium (Bio-optica 05-1740: Milan, Italy) [20,21,22,24,25].

### 2.11. Human Cell Cultures

THLE2 human hepatic cell line (American Type Culture Collection, ATCC) was cultured in bronchial epithelial cell basal medium (BEBM) supplemented with a growth factors BulleKit (Lonza/Clonetics Corporation, Walkersville, MD, USA), 70 ng/mL phosphoethanolamine, 5 ng/mL epidermal growth factor, 10% (*v*/*v*) FBS, and 1% (*v*/*v*) Glutamine–Penicillin–Streptomycin solution (MERCK; Taufkirchen, Germany). THLE-2 cells were grown on culture plates pre-coated with a mixture of 0.01 mg/mL fibronectin (#33010018, Sigma Aldrich, St. Louis, MO, USA), 0.01 mg/mL bovine serum albumin (#A4503, Sigma Aldrich, St. Louis, MO, USA), and 0.03 mg/mL collagen type I (#sc-136157, Santa Cruz, Dallas, TX, USA). Cells were tested for mycoplasma contamination.

#### 2.11.1. Cell Transfection

In the study, 0.5 × 105 THLE2 cells were seeded in a twenty-four-well plate, and cultured 24 h before transfection. Transfections were performed using Dharmafect 1 reagent (T-2001-03, Dharmacon Lafayette, CO, USA), following the manufacturer’s protocol. Briefly, 50 pmol of each siRNA was diluted in 200 μL of OptiMEM (#31985-070, Life Technologies, Carlsbad, CA, USA) and mixed with 6.5 μL of Dharmafect 1, pre-diluted in 193.5 μL of OptiMEM. The final mixture (500 μL) was added to each well. After six hours, the transfection medium was replaced by fresh BEGM, and the corresponding treatments were applied. Cells were transfected with specific small-interference RNA (si-RNA) to knock down the expression of OR4M1 (L-032481-02-0010, Dharmacon, Lafayette, CO, USA) or with non-targeting siRNA used as a negative control (Dharmacon, Lafayette, CO, USA).

#### 2.11.2. Resveratrol Treatment

THLE2 cells silencing OR4M1 were incubated in a medium supplemented with 1 μg/mL resveratrol (R5010, MERCK, Taufkirchen, Germany), a pharmacological activator of SIRT1. The dose was selected based on da Silva Lima et al. (PMID: 34555423), ensuring effective SIRT1 activation while maintaining cell viability. After 24 h, cells were either collected for mRNA extraction or subjected to Oil Red O staining.

#### 2.11.3. Oil Red O Staining

Cells were seeded on sterile glass coverslips placed in the wells of a 24-well plate. Following treatment, the culture medium was removed, and cells were gently rinsed with phosphate-buffered saline (PBS). Fixation was carried out using 10% neutral buffered formalin for 10 min at room temperature. After fixation, wells were washed with PBS, and 200 µL of freshly prepared working Oil Red O solution was added to each well. Cells were incubated for 20 min at room temperature, followed by thorough washing with distilled water until the background was clear. Subsequently, a counterstaining step was performed by incubating the coverslips with Mayer’s hematoxylin for 10 min, after which they were rinsed again with distilled water. Coverslips were then carefully removed and mounted cell-side down onto glass microscope slides using an aqueous mounting medium. Stained preparations were examined under a light microscope to evaluate intracellular lipid droplet accumulation. In these histological staining techniques, up to 6 representative microphotographs of cells were taken with a BX51 microscope equipped with a DP70 digital camera (Olympus, Tokyo, Japan). Lipids in Oil Red O-stained sections were quantified using ImageJ 1.52p software.

### 2.12. Patients

Liver specimens were obtained from a cohort of 21 patients with severe obesity undergoing bariatric surgery at the Clínica Universidad de Navarra. Obesity was defined as a body mass index (BMI) ≥ 30 kg/m^2^ and a body fat percentage (BF) ≥ 35%, with BMI calculated as weight (kg) divided by height squared (m^2^), and BF assessed via air-displacement plethysmography (Bod-Pod, Life Measurements, Concord, CA, USA). Participants were categorized into normoglycemia (NG) or T2DM subgroups according to the diagnostic criteria established by the Expert Committee on the Diagnosis and Classification of Diabetes. Inclusion criteria required a comprehensive clinical evaluation, including physical examination, laboratory profiling, abdominal ultrasonography, and histological confirmation of metabolic MASLD via percutaneous liver biopsy. Biopsies were assessed by a hepatopathologist blinded to clinical and biochemical data, applying the Kleiner and Brunt criteria to derive the MASLD activity score (NAS), which integrates scores for steatosis, lobular inflammation, and hepatocellular ballooning (range 0–8).

Exclusion criteria included the following: (a) alcohol consumption exceeding 20 g/day for females or 30 g/day for males; (b) serological evidence of hepatitis B surface antigen or hepatitis C virus antibodies in the absence of prior immunization; (c) current or prior use of hepatotoxic agents associated with MASLD (e.g., amiodarone, valproate, tamoxifen, methotrexate, corticosteroids, or antiretroviral therapy); and (d) alternative liver pathologies, including autoimmune hepatitis, hereditary hemochromatosis, Wilson’s disease, or α-1-antitrypsin deficiency. Individuals with T2DM were excluded if they were receiving insulin or medications known to affect endogenous insulin production. All T2DM cases had a disease duration of less than 2–3 years or were newly diagnosed based on clinical anamnesis and metabolic parameters. The study protocol adhered to the Declaration of Helsinki (2013 revision) and received approval from the Institutional Ethics Committee (protocol no. 2021.005, approved on 11 February 2021). All participants provided written informed consent prior to enrollment.

### 2.13. Data Analysis and Statistics

Data are presented as mean ± SEM for each genotype. Protein and mRNA expression levels were expressed as percentages relative to the control group (either fed ad libitum or treated with shGFP). Statistical analyses were conducted based on the number of groups and the distribution of the data. Comparisons between two groups were analyzed using either the Student’s *t*-test or the Mann–Whitney U test, depending on the normality of the data distribution. For comparisons involving more than two groups, two-way ANOVA or the Kruskal–Wallis test was applied, followed by Bonferroni post hoc analysis. A *p*-value < 0.05 was considered statistically significant. The normality of the data was assessed using the Shapiro–Wilk test. The sample size for each group was calculated based on previous studies, and some data points were eliminated from the study after performing outlier analysis calculation. All analyses were performed using Excel (Microsoft Corp., Redmond, WA, USA) and GraphPad PRISM 10 software.

## 3. Results

### 3.1. Olfr734 Is Involved in the Adaptation of Glucose Production to Nutrient Availability

**Figure 1:**
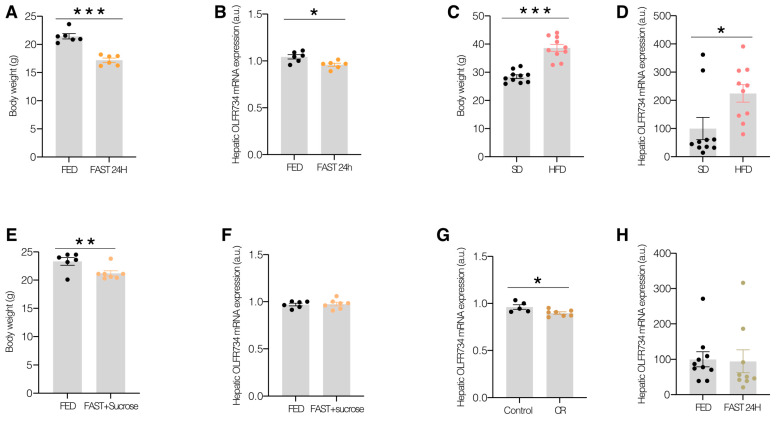
Olfr734 is involved in the adaptation of glucose production to nutrient availability. (**A**) Body weight of mice with different nutritional statuses (fed and fast 24 h) under standard diet. (**B**) Hepatic mRNA levels of Olfr734 in the liver of mice with different nutritional statuses (fed and fast 24 h). (**C**) Body weight of DIO mice. (**D**) Hepatic mRNA levels of Olfr734 in the liver of DIO mice. (**E**) Body weight and (**F**) hepatic mRNA levels for Olfr734 after ad libitum feeding (as a control) vs. 24 h fast followed by sugar feeding and (**G**) after ad libitum feeding vs. caloric restriction (CR) and (**H**) with different nutritional statuses (fed and fast 24 h) in DIO mice; expression of β-actin serving as loading control. Values are mean ± SEM of 4–11 animals per group. * *p* ≤ 0.05, ** *p* ≤ 0.01, *** *p* ≤ 0.001 vs. controls (two-tailed unpaired *t*-test).

To examine the regulation of hepatic Olfr734 in relation to nutritional status, we quantified the levels of this receptor in lean mice subjected to a 24 h fasting period and compared these levels with a control group of mice fed ad libitum. As anticipated, mice that fasted for 24 h exhibited a significant reduction in glucose levels (Figure S1A) and body weight compared to the ad libitum-fed animals (Figure 1A).

We subsequently evaluated the hepatic mRNA Olfr734 levels in these animals and observed a significant reduction after 24 h of fasting compared to mice fed ad libitum (Figure 1B). We then examined the Olfr734 levels in conditions of excessive nutrient intake, such as in DIO mice. As expected, there was a significant increase in the glucose levels (Figure S1B) and body weight of DIO animals compared to those on a control STD (Figure 1C). Furthermore, the liver Olfr734 levels in DIO mice increased after 10 weeks of an HFD compared to STD-fed animals (Figure 1D), suggesting that Olfr734 is a sensitive signal, responsive to changes in nutritional status induced by both short- and long-term variations in nutrient availability. Given that previous data have shown that Olfr734 inhibition reduces hepatic glucose production, along with the role of asprosin in maintaining glucose homeostasis [11], we sought to investigate whether Olfr734 might be involved in the adaptation of glucose production to nutrient availability. To explore this, and considering the regulation of Olfr734 in the liver following short-term changes in nutritional status such as fasting, we conducted an experiment where one group of animals was fasted and fed with sugar cubes, while another group was fed ad libitum with an STD. Notably, we found that sugar intake normalizes the fasting-induced reduction in Olfr734 levels (Figure 1F). Sugar-fed fasted mice exhibited blood glucose levels similar to those of mice fed ad libitum (Figure S1C); however, they experienced significantly greater weight loss than the control ad libitum-fed group (Figure 1E). To further investigate this regulatory mechanism, we examined the effect of another model of hypoglycemia, that is, calorie restriction (CR) (Figure S1D). Consistent with our findings, a 60% CR induces a decrease in hepatic Olfr734 levels in mice compared to the ad libitum-fed animals (Figure 1G).

Upon obtaining clear results indicating a reduction in Olfr734 levels under hypoglycemic conditions resulting from fasting or caloric restriction (CR), we proceeded to examine the role of Olfr734 mRNA expression during fasting in states of excessive nutrient intake, such as DIO. Interestingly, while the glucose levels decreased after fasting in DIO mice, the expression of this receptor did not decrease as observed during fasting on an STD (Figure 1H). These findings putatively suggest that Olfr734 functions as a sensor of hepatic glucose availability, although its regulatory capacity may be compromised in metabolically challenged states such as obesity. This characterizes our target as being able to signal sensitively and responsively to glucose level variations induced by changes in nutritional status.

### 3.2. Specific Olfr734 Knockdown Increases Hepatic Lipid Content in DIO Mice

**Figure 2:**
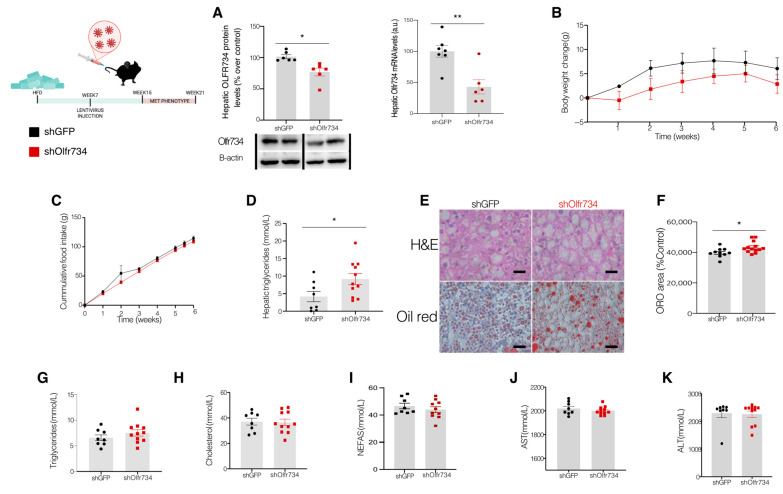
Specific Olfr734 knockdown increases hepatic lipid content in DIO mice. (**A**) Protein levels and mRNA levels of Olfr734 in the liver of male mice injected with scrambled or shOlfr734 lentiviruses. (**B**) Effect of Olfr734 in the liver of male mice injected with scrambled or shOlfr734 lentiviruses on body weight (**C**) food intake, (**D**) Liver triglycerides, (**E**) Liver cell histologies: bar scale size represents 100 µm, (**F**) Oil Red Area quantification, (**G**) plasma triglycerides, (**H**) plasma cholesterol, (**I**) plasma NEFAS, (**J**) plasma AST, and (**K**) ALT. β-actin was used as a housekeeping. Dividing lines indicate spliced bands from the same gel. Values are mean ± SEM of 6–11 animals per group. * *p* ≤ 0.05, ** *p* ≤ 0.01 vs. controls (two-tailed unpaired *t*-test and two-way ANOVA).

Given our findings that Olfr734 expression in the liver of WT animals is elevated in obesity, we aimed to investigate the effects of specific Olfr734 knockdown in the liver of DIO mice. We opted to target Olfr734 rather than asprosin, as the latter is primarily secreted by WAT, with minimal levels reported in the liver [14,18]. To achieve this, we employed virogenetic techniques to inhibit Olfr734 in the livers of WT mice fed an HFD for 15 weeks. The mice were fed an HFD for 8 weeks, after which one group received a scrambled lentivirus encoding GFP (shGFP), while another group was injected with a lentivirus targeting Olfr734 (shOlfr734) via the tail vein. The effectiveness of the injection was assessed by measuring the Olfr734 protein and mRNA levels in the liver. We observed a significant decrease in the Olfr734 levels in the shOlfr734-treated mice compared to those injected with shGFP with both approaches (Figure 2A).

Importantly, the effect of the lentivirus seems to be restricted to the liver as the protein levels of Olfr734 in other tissues such as the WAT, hypothalamus, or cortex show no variations (Figure S2). Our data indicate that both groups of animals had similar body weight and food intake during the course of the experiment (Figure 2B,C). However, we sought to determine the role of hepatic Olfr734 inhibition in DIO animals with respect to hepatic fat content, the main driver of MASLD. Unexpectedly, it was found that animals injected with shOlfr734 displayed a significant increase in TG content in the liver compared to the control animals (Figure 2D). To determine the complete lipid profile, we measured the circulating levels of TG, cholesterol, and NEFAS from plasma (Figure 2E–G). Our measurements indicated that there were no differences in the levels of these parameters between mice injected with a scrambled virus and the group injected with shOlfr734. Finally, to determine possible differences in the hepatic damage caused by the injection of shOlfr734, we measured the levels of hepatic transaminases in plasma. However, no differences were found between the two groups (Figure 2H,I).

### 3.3. Olfr734 Knockdown Inhibits the SIRT1/ER Stress Signaling Pathway

**Figure 3:**
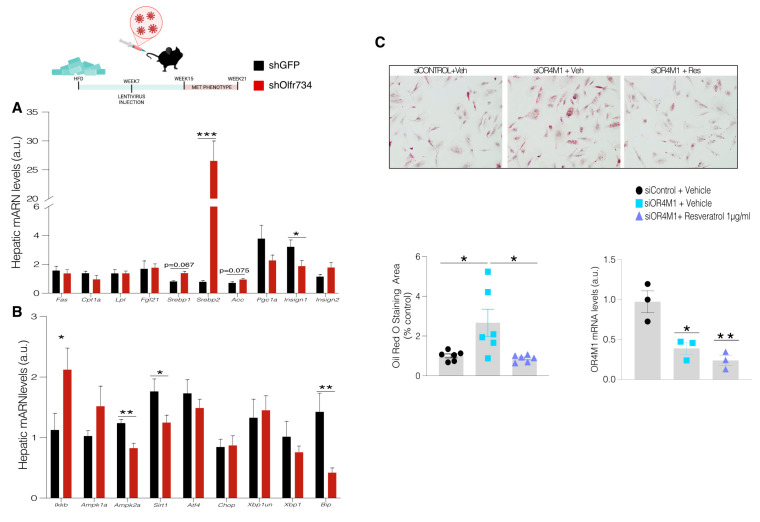
Olfr734 knockdown inhibits the SIRT1/ER stress signaling pathway. (**A**) Liver mRNA levels of fatty acid metabolism enzymes: (**B**) inflammatory markers and Ampkα1 and α2, Sirt1, Ikkβ, Atf4, Chop, Xbp1un, Xbp1 total, Bip in male DIO mice injected with shOlfr734 or GFP scrambled lentiviruses. Expression of β-actin serving as loading control. (**C**) THLE-2 cells silencing OR4M1 incubated with 1 μg/mL resveratrol. Values are mean ± SEM of 6–8 animals/wells per group. * *p* ≤ 0.05, ** *p* ≤ 0.01, *** *p* ≤ 0.001 vs. controls (two-tailed unpaired *t*-test or one-way ANOVA).

To investigate the precise signaling pathway mediating the effects of Olfr734 on hepatic lipogenesis, we performed some molecular analyses on the livers of both animal groups. We observed a clear trend of increased levels of the adipogenic markers, namely Acetyl-CoA carboxylase and sterol regulatory element-binding protein 1 as well as a significant increase in the levels of sterol regulatory element-binding protein 2 and a reduction in the levels of insulin-induced gene 1. In addition, the levels of the inflammatory marker inhibitory kappa B kinase beta (Ikkβ) were also increased [28] (Figure 3A).

Our data indicate that Olfr734 expression varies in response to changes in nutrient availability, prompting us to evaluate the expression of two well-known nutritional sensors, AMP-activated protein kinase (AMPK) and sirtuin 1 (Sirt1). The levels of AMP-activated protein kinase α2 subunit (Ampkα2) were significantly reduced in the livers of shOlfr734-injected animals (Figure 3B). This finding aligns with previous data linking asprosin signaling and MASLD [17]. Similarly, we observed a reduction in the levels of Sirt1, consistent with several reports demonstrating that this deacetylase is upregulated in fasting conditions and reduced in obesity [29]. Notably, one report showed reduced levels of Sirt1 in the liver of constitutive knockdown mice, which are obese and present MASLD, for the isoform TAp63 [22]. To gain deeper insight into these mechanisms and to determine the molecular underpinnings of the effects of Olfr734 inhibition on MASLD, we evaluated the role of the endoplasmic reticulum (ER) stress pathway in our experimental setting. The inflammatory marker Ikkβ has been reported to link ER stress, inflammation, and liver disease [27]. Moreover, several studies have indicated that ER stress increases lipogenesis, and the unfolded protein response is usually activated in states of positive energy balance, such as obesity or liver diseases [27,28,30,31]. However, no previous data have linked the actions of asprosin–Olfr734 signaling to this pathway. We measured the expression of Activating Transcription Factor 4 (Atf4), CCAAT-enhancer-binding protein homologous protein (Chop), and X-box binding protein 1 (Xbp1), but did not find any variations in these ER stress markers (Figure 3B). Despite this, we found a downregulation of the glucose-regulated protein 78/binding immunoglobulin protein (Grp78/BiP), a chaperone reported to attenuate ER stress [25,27,30] (Figure 3B). This finding aligns with the fact that overexpression of Grp78/BiP in the liver and hypothalamus of DIO mice has been used therapeutically to treat MASLD and obesity by reducing adiposity and fat content in the liver [25,27,30]. It can therefore be suggested that the molecular underpinnings triggering lipid storage in the liver seem to include the activation of ER stress-related pathways, favoring lipid storage. Interestingly, the action of shOlfr734 on the liver seems to be specific to this pathway as the levels of other relevant nutritional or energy sensors in hepatic tissue such as mTOR, FGF21, GDF15, or ANGPTL4 showed no variation between groups (Figure S3).

In order to gain mechanistical insight into our experiments, we performed a functional study in THLE-2 cells. Thus, we initially checked if siRNAs directed against OR4M1 and transfected into THLE-2 cells might affect lipid metabolism under basal conditions. Under these circumstances, siRNA OR4M1 increases lipid accumulation (Figure 3C). However, when cells were co-transfected with resveratrol (a Sirt1 activator) and siRNA OR4M1, we found amelioration in OR4M1-induced lipid accumulation. These data suggest that the effects of OR4M1 on the liver with regard to lipid metabolism are mediated by Sirt1.

### 3.4. Specific Hepatic Olfr734 Knockdown Promotes Hepatic Glucose Production in DIO Mice

**Figure 4:**
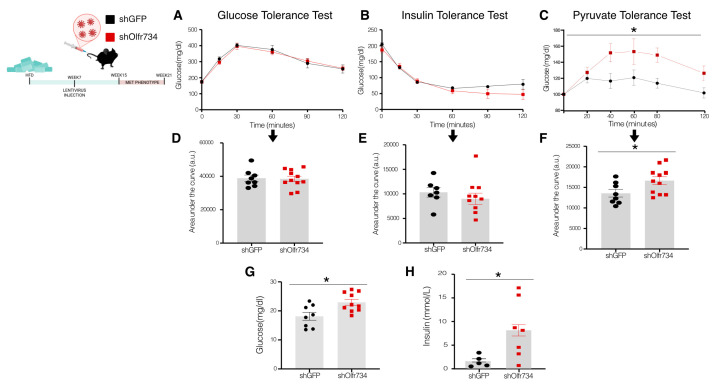
Specific hepatic Olfr734 knockdown promotes hepatic glucose production in DIO mice: (**A**,**D**) glucose tolerance test and area under the curve; (**B**,**E**) insulin tolerance test and area under the curve; (**C**,**F**) pyruvate tolerance test and area under the curve; (**G**) levels of glucose and (**H**) insulin in male DIO mice injected with shOlfr734 or GFP scrambled lentiviruses. Values are mean ± SEM of 8–11 animals per group. * *p* ≤ 0.05 vs. controls (paired *t*-test and two-way ANOVA).

Given that obesity is characterized by insulin resistance and insulin plays a crucial role in maintaining glucose homeostasis, we aimed to investigate the specific impact of hepatic Olfr734 knockdown on this parameter in DIO mice. To this end, we utilized the same cohort of animals as in the previous experiment, in which Olfr734 expression was specifically genetically inhibited in the liver of DIO mice. Initially, we conducted a series of tolerance tests for insulin, glucose, and pyruvate. Our findings indicated no significant differences in glucose tolerance or insulin sensitivity, as assessed by GTT and ITT, between the control group and the group receiving shOlfr734 injections (Figure 4A,B).

Upon conducting a PTT, it was observed that animals treated with shOlfr734 exhibited elevated glucose levels, suggesting an enhanced gluconeogenic capacity (Figure 4C). Furthermore, we assessed the levels of circulating insulin and glucose. Our findings indicate that both the glucose and insulin levels were elevated in the liver of our specific knockdown model (Figure 4C,D), suggesting that the shOlfr734-injected animals have increased hepatic insulin resistance compared to the control animals injected with shGFP.

### 3.5. Specific Hepatic Olfr734 Knockdown Induces Glucose Production During Fasting

**Figure 5:**
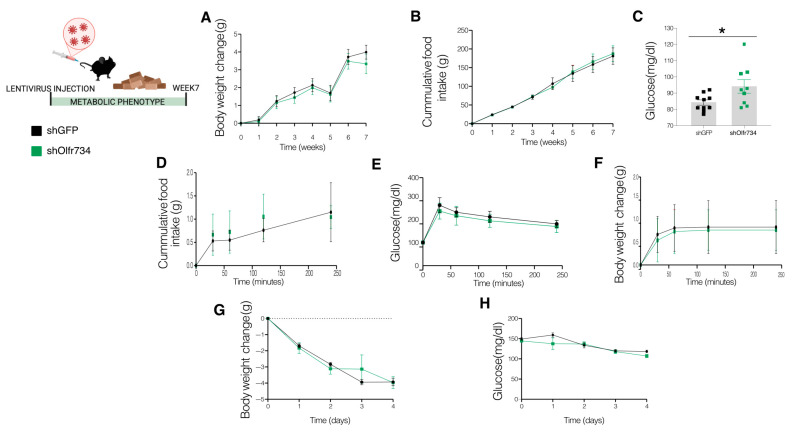
Specific hepatic Olfr734 knockdown triggers glucose production in fasting. (**A**) Body weight, (**B**) food intake, (**C**) blood glucose levels after 24 h fasting and postprandial, (**D**) food intake, (**E**) glucose levels and (**F**) body weight change in male mice injected with shOlfr734 with standard diet, and (**G**) body weight change and (**H**) blood glucose levels of male mice subjected to 60% CR and injected with shOlfr734 with standard diet (*n* = 9–10 per group). * *p* ≤ 0.05 vs. controls (two-tailed unpaired *t*-test and two-way ANOVA).

Given that Olfr734 expression is modulated by nutritional status and plays a role in adjusting glucose production to nutrient availability, we sought to investigate the function of endogenous Olfr734 in glucose metabolism. To this end, we developed a mouse model with hepatic deletion of Olfr734 by administering shOlfr734 via the tail vein to WT mice maintained on an STD. These animals had similar body weight and food intake to their respective controls injected with shGFP (Figure 5A,B).

The subsequent phase involved subjecting the animals to a 24 h fasting period. At this juncture, it was observed that mice injected with shOlfr734 exhibited significantly elevated blood glucose levels after 24 h of fasting compared to their respective controls injected with shGFP (Figure 5C). To investigate the hepatic role of Olfr734 in postprandial glucose regulation in greater detail, we assessed the model’s capacity to restore glucose levels following nutrient ingestion. For this purpose, the mice underwent overnight fasting and were subsequently refed with an STD for four hours. We analyzed potential changes in food intake, body weight, and glucose levels during this period; however, our findings indicated no changes in any of the parameters studied (Figure 5D–F). To shed further light on the phenotype of our conditional Olfr734 knockdown mouse model, we implemented a 60% caloric restriction on the same cohort of animals and monitored body weight and glucose levels over four consecutive days. Contrary to our expectations, both groups of animals demonstrated similar body weight and blood glucose levels (Figure 5G,H).

### 3.6. Hepatic OR4M1 Levels, a Human Ortholog of Mice Olfr734, Are Increased in Subjects with T2DM in a Sex-Dependent Manner

**Figure 6:**
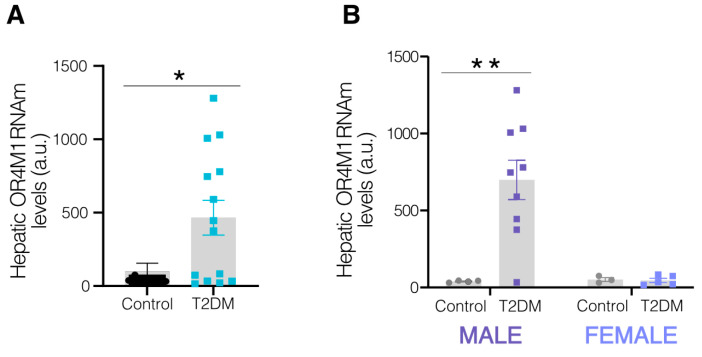
Hepatic OR4M1 levels are increased in subjects with T2DM in a sex-dependent manner. (**A**) Hepatic OR4M1 levels are associated with human type 2 diabetes (T2DM). (**B**) Hepatic OR4M1 mRNA levels in patients with normoglycemia (NG) or type 2 diabetes in males and females (T2DM). Expression of β-actin serving as loading control, and control values were normalized to 100%. (n = 4–14 per group). Data are presented as mean ± SEM; * *p* < 0.05 and ** *p* < 0.01 vs. control (two-tailed unpaired *t*-test). One data point from the control group was discarded after performing an outlier calculation.

Circulating levels of asprosin are increased in patients with MASLD, chronic liver disease, and diabetes. In considering these observations, we questioned whether the regulation of Olfr734 levels in our mouse models could be extrapolated to human subjects. To investigate this, we measured the levels of the human gene encoding the asprosin receptor, OR4M1, in the livers of patients with obesity, both normoglycemic and with T2DM. Our results showed that the levels of this receptor were elevated in the livers of patients with obesity and T2DM compared to the controls with obesity and normoglycemia (Figure 6A). The dataset was further scrutinized, with analyses conducted separately in men and women. Interestingly, when we split the data according to sex, it was found that there was an increase in the levels of OR4M1 in the livers of men but not in women (Figure 6B). These findings suggest that increased OR4M1 levels are associated with hyperglycemia in humans in a sex-dependent manner.

## 4. Discussion

MASLD is a globally prevalent condition with limited effective pharmacological interventions available. Understanding its mechanism of action and identifying new therapeutic targets are urgently needed. Among the numerous molecules implicated in the pathophysiology of MASLD is the recently identified hormone asprosin, which serves as the endogenous ligand for the olfactory receptor Olfr734. Asprosin binds to and activates the Olfr734 receptor, stimulating food intake and promoting hepatic glucose production. Significantly, circulating asprosin levels are elevated in obesity and other metabolic disorders, such as T2DM and MASLD. Inhibition of asprosin has been proposed as a potential treatment for MASLD in rodent models. Despite the hormone’s involvement in MASLD and gluconeogenesis, few studies have explored the specific role of Olfr734 in liver function. The Olfr734 receptor is predominantly expressed in the liver, a key metabolic organ responsible for maintaining glucose and lipid homeostasis. In this study, we chose to target the Olfr734 receptor rather than asprosin itself, given the reported reduced presence of this endocrine factor in the liver compared to other organs. Indeed, there is limited information in the literature regarding the physiological modulation of this receptor and its potential metabolic consequences. The main findings of our study are as follows: (1) Olfr734 levels in the liver may serve as a sensor of nutrient and glucose availability; (2) Olfr734 knockdown in the liver exacerbates MASLD and increases hepatic glucose production in DIO mice; (3) the effects of Olfr734 knockdown in the liver inhibit the Sirt1/ER stress pathway; and (4) the hepatic levels of Olfr734 are upregulated in patients with T2DM in a sex-dependent manner.

In this study, we observed that the hepatic expression of this receptor may serve as an indicator responsive to varying nutritional states, exhibiting a distinct expression pattern from asprosin. Our findings indicate that Olfr734 expression is downregulated during fasting, while it is upregulated in obesity. Interestingly, prior research has not quantified the levels of this receptor in the liver under these experimental conditions. Furthermore, the expression pattern of Olfr734 in the liver during fasting contrasts sharply with that of plasma asprosin. However, in obesity, the levels of Olfr734 and asprosin are similarly elevated. This phenomenon may be attributed to the substantial release of asprosin into the bloodstream during fasting, potentially leading to the downregulation or desensitization of its hepatic receptor (e.g., through endocytosis) as a compensatory response to elevated circulating asprosin levels. A counterregulatory mechanism may be activated in the liver, resulting in the inhibition of Olfr734 expression and thus contributing to this specific phenotype. Collectively, these findings suggest a potential metabolic role for Olfr734. While asprosin is known to regulate MASLD in both mice and humans, the precise role of its receptor, Olfr734, remains unclear. The elevated levels of Olfr734 in obesity prompted us to hypothesize that the targeted inhibition of Olfr734 in the liver could be a promising strategy for improving MASLD treatment. Contrary to our expectations, however, this approach exacerbated MASLD. This unexpected and counterintuitive result lacks a clear explanation. It is plausible to speculate that an as-yet-undiscovered ligand (antagonist) for Olfr734 may be involved, akin to the recently identified endogenous antagonist for the GHSR1a–ghrelin system, LEAP-2 [32,33,34]. Another possibility is that a compensatory effect exists in the action of Olfr734 inhibition on the liver. Thus, the liver, in order to counteract pathways that repress lipid storage, would increase the levels of TG in the liver in response. Nevertheless, the function of this receptor is poorly understood and may be compensated by other receptors or pathways.

A key objective of the current study was to investigate the downstream molecular pathways controlling the hepatic actions of Olfr734 knockdown. We could reproduce previous findings linking the actions of asprosin and the pathways related to fatty acid metabolism and inflammation. Interestingly, we found new molecular underpinnings for the induction of MASLD by Olfr734 knockdown. Our data indicate that the Olfr734 inhibition-induced hepatic fat content inhibits Sirt1/ER stress-associated pathways. In fact, this study is the first to report the link between these molecular pathways and the action of asprosin signaling.

The observed downregulation of Sirt1 in DIO animals injected with shOlfr734 accords with the established regulation of this deacetylase in obesity. It is noteworthy that hepatic Sirt1 expression increases during nutrient deprivation and decreases in obesity, particularly in peripheral tissues such as the liver of DIO mice, which are often affected by MASLD [29,35,36,37]. Sirt1 plays a crucial role in regulating hepatic lipid metabolism, preventing lipid accumulation and the induction of adipogenesis enzymes [38]. The specific deletion of hepatic Sirt1 impairs the fatty acid β-oxidation pathway, thereby heightening the susceptibility of mice to HFD-induced dyslipidemia, hepatic steatosis, inflammation, and ER stress [39,40]. This finding is consistent with another study demonstrating that the deletion of hepatic Sirt1 leads to the development of liver steatosis [41]. Moreover, we demonstrate the involvement of Sirt1 in the effect of OR4M1 inhibition in the liver by functional experiments in THLE-2 cells. In addition to these findings, we observed an increase in the inflammatory marker Ikkβ, which has been previously reported to be upregulated in the liver of obese animals with MASLD [27]. Notably, Ikkβ has been proposed as the molecular link between inflammation and ER stress. We investigated this pathway to identify a new mechanism of action that might explain our findings. Accumulating evidence has demonstrated a strong association between ER stress and metabolic diseases such as obesity and T2DM, as well as liver diseases [25,27,30,42,43]. It has been observed that improving protein folding, for instance, through chemical chaperones, ameliorates obesity and MASLD [25,27,30,42,43]. In this context, Bip is a chaperone that modulates protein folding in response to cellular stress. Its overexpression in the hypothalamus and liver of DIO animals has been consistently reported to diminish obesity and MASLD [25,27,30]. Our data are confirmed by these previous findings, and although we did not observe an increase in ER stress marker levels, namely Chop, Xbp1, or Atf4, we found a significant reduction in Bip levels in the livers of our DIO mice injected with shOlfr734.

Gluconeogenesis also contributes to hyperglycemia in T2DM, and these patients exhibit a defect in insulin action, a condition known as insulin resistance, which may impact hepatic glucose regulation. Furthermore, ER stress is closely associated with obesity-related insulin resistance in peripheral tissues, such as the liver [42,43]. Interestingly, the phenotype of our mice injected with shOlfr734 is characterized by an increase in insulin secretion, suggesting potential insulin resistance. This observation aligns with the phenotype described here, as insulin resistance is a critical component of obesity and MASLD. In addition, we observed that, alongside insulin resistance, hepatic glucose production in these mice increased, another key function of asprosin–Olfr734 signaling. Moreover, the knockdown of Olfr734 in lean animals results in elevated glucose levels after 24 h of fasting in mice fed ad libitum, contrary to previous data indicating the opposite [14]. However fasting and caloric restriction did not uniformly impact glucose metabolism in Olfr734-knockdown mice. This discrepancy might be due to the fall in glucose levels in CR not being as great in fasted mice and, as a result, Olfr734 inhibition does not induce an effect as we found in fasting conditions. We currently lack a clear explanation for this phenomenon, necessitating further research to elucidate the precise role of asprosin signaling in hepatic function. Our data suggest that the Olfr734 levels are low in hypoglycemic states such as fasting or CR and high in hyperglycemic states such as obesity. Furthermore, the reduction in receptor expression observed during fasting is attenuated in fasting animals fed with sucrose pellets. It is noteworthy that these animals exhibit similar glucose levels to the ad libitum-fed group but differ in caloric/nutrient intake and have lower body weight. These findings suggest that, in addition to serving as a nutrient sensor, our target is a signal sensitive and responsive to variations in glucose levels.

In an effort to ascertain the translational relevance of our findings, we assessed the levels of our target in the livers of human patients with diabetes. Our analysis revealed that the mRNA levels of OR4M1 are elevated in patients with obesity and T2DM compared to those with obesity and normoglycemia. Given the significant influence of gender on liver disease and various metabolic pathway disorders, we stratified our data by sex to determine whether OR4M1 expression exhibits a similar pattern in both sexes. Our findings indicate a pronounced sexual dimorphism in the action of OR4M1. However, it is important to note that one limitation in our study is the small patient sample size that impedes detecting a positive or negative correlation of OLFR734 levels and biochemical and anthropometric measurements from these patients. In conclusion, this study is the first to (i) investigate the modulation of Olfr734 in the development of MASLD; (ii) establish a link between asprosin signaling-induced liver steatosis and a Sirt/ER stress pathway; and iii) associate the hepatic levels of OR4M1 with patients with T2DM.

Because Olfr734 is involved in the pathophysiology of different diseases associated with obesity, such as T2DM and MASLD, identifying the molecular mechanisms underlying these disorders will help identify more effective methods for their prevention and treatment. In this study, our first aim was to discover a new therapeutic target by manipulating the Olfr734 levels. If we consider that the levels of Olfr734 in the liver of DIO mice and T2DM patients are higher than those in the controls, the development of a specific pharmacological antagonist/inverse agonist for Olfr734 or another system by which the expression of the Olfr734 gene could be genetically inhibited specifically in the liver in a safe and effective manner without side effects could be a priori a conceivable option. However, our results do not support this possibility, probably because of compensatory mechanisms in the action of Olfr734 inhibition in the liver, precluding the use of these approaches to treat these metabolic diseases. Despite this, we believe that this work provides important insights into the role of Olfr734 in MAFLD and T2DM.

Given that asprosin was only identified nine years ago, limited information is available regarding the regulation and mechanism of Olfr734 action. Substantially more research is required to elucidate the biology of this endocrine signaling system.

## Data Availability

The patients’ data reported in this study cannot be deposited in a public repository because they were used with permission for this study with restrictions that do not allow for the data to be redistributed or made publicly available. This paper does not report the original code. Any additional information required to respond to any question related to the data reported in this paper is available from the lead contact upon request.

## Supplementary Materials

The following supporting information can be downloaded at: https://www.mdpi.com/article/10.3390/nu17152426/s1, Figure S1: Glucose levels of different models used in Figure 1, Values are mean ± SEM, ** *p* < 0.01 and *** *p* < 0.001 vs. control (two-tailed unpaired *t*-test); Figure S2: OLFR734 protein levels in WAT, cortex, and hypothalamus. β-actin and vinculin were used as a housekeeping. Dividing lines indicate spliced bands from the same gel. Values are mean ± SEM of seven samples per group. Figure S3: Hepatic protein levels of ANGPTL4, GDF15, FGF21, mTOR, and pmTOR. β-actin was used as a housekeeping. Dividing lines indicate spliced bands from the same gel. Values are mean ± SEM of seven samples per group. Table S1: List of primers used.

## References

[1] Eslam M., Sanyal A.J., George J. (2020). International Consensus Panel. MAFLD: A Consensus-Driven Proposed Nomenclature for Metabolic Associated Fatty Liver Disease. Gastroenterology.

[2] Angulo P. (2002). Nonalcoholic fatty liver disease. N. Engl. J. Med..

[3] Harrison S.A., Gawrieh S., Roberts K., Lisanti C.J., Schwope R.B., Cebe K.M., Paradis V., Bedossa P., Whitehead J.M.A., Labourdette A. (2021). Prospective evaluation of the prevalence of non-alcoholic fatty liver disease and steatohepatitis in a large middle-aged US cohort. J. Hepatol..

[4] Pagano G., Pacini G., Musso G., Gambino R., Mecca F., Depetris N., Cassader M., David E., Cavallo-Perin P., Rizzetto M. (2002). Nonalcoholic steatohepatitis, insulin resistance, and metabolic syndrome: Further evidence for an etiologic association. Hepatology.

[5] Younossi Z.M., Golabi P., Paik J.M., Henry A., Van Dongen C., Henry L. (2023). The global epidemiology of nonalcoholic fatty liver disease (NAFLD) and nonalcoholic steatohepatitis (NASH): A systematic review. Hepatology.

[6] Estes C., Anstee Q.M., Arias-Loste M.T., Bantel H., Bellentani S., Caballeria J., Colombo M., Craxi A., Crespo J., Day C.P. (2018). Modeling NAFLD disease burden in China, France, Germany, Italy, Japan, Spain, United Kingdom, and United States for the period 2016–2030. J. Hepatol..

[7] Targher G., Corey K.E., Byrne C.D., Roden M. (2021). The complex link between NAFLD and type 2 diabetes mellitus—mechanisms and treatments. Nat. Rev. Gastroenterol. Hepatol..

[8] Qu W., Ma T., Cai J., Zhang X., Zhang P., She Z., Wan F., Li H. (2021). Liver Fibrosis and MAFLD: From Molecular Aspects to Novel Pharmacological Strategies. Front. Med..

[9] Attia S.L., Softic S., Mouzaki M. (2021). Evolving Role for Pharmacotherapy in NAFLD/NASH. Clin. Transl. Sci..

[10] Yoneda M., Honda Y., Saito S., Nakajima A. (2021). What considerations are there for the pharmacotherapeutic management of nonalcoholic steatohepatitis?. Expert Opin. Pharmacother..

[11] Romere C., Duerrschmid C., Bournat J., Constable P., Jain M., Xia F., Saha P.K., Del Solar M., Zhu B., York B. (2016). Asprosin, a Fasting-Induced Glucogenic Protein Hormone. Cell.

[12] Liu L.J., Kang Y.R., Xiao Y.F. (2021). Increased asprosin is associated with non-alcoholic fatty liver disease in children with obesity. World J. Pediatr..

[13] Cui J., Liu Y., Li M., Yin J., Yang J., Xu L. (2024). Association of serum asprosin with metabolic dysfunction-associated fatty liver disease in older adult type 2 diabetic patients: A cross-sectional study. BMC Endocr. Disord..

[14] Liu Y., Long A., Chen L., Jia L., Wang Y. (2020). The Asprosin-OLFR734 module regulates appetitive behaviors. Cell Discov..

[15] Li E., Shan H., Chen L., Long A., Zhang Y., Liu Y., Jia L., Wei F., Han J., Li T. (2019). OLFR734 Mediates Glucose Metabolism as a Receptor of Asprosin. Cell Metab..

[16] Ke F., Xue G., Jiang X., Li F., Lai X., Zhang M., Shen Y., Gao L. (2020). Combination of asprosin and adiponectin as a novel marker for diagnosing non-alcoholic fatty liver disease. Cytokine.

[17] Wang C., Zeng W., Wang L., Xiong X., Chen S., Huang Q., Zeng G., Huang Q. (2024). Asprosin aggravates nonalcoholic fatty liver disease via inflammation and lipid metabolic disturbance mediated by reactive oxygen species. Drug Dev. Res..

[18] Zhang B., Lu J., Jiang Y., Feng Y. (2023). Asprosin contributes to nonalcoholic fatty liver disease through regulating lipid accumulation and inflammatory response via AMPK signaling. Immun. Inflamm. Dis..

[19] Lu Y., Yuan W., Xiong X., Huang Q., Chen S., Yin T., Zhang Y., Wang Z., Zeng G., Huang Q. (2023). Asprosin aggravates vascular endothelial dysfunction via disturbing mitochondrial dynamics in obesity models. Obesity.

[20] Quiñones M., Hernández-Bautista R., Beiroa D., Heras V., Torres-Leal F.L., Lam B.Y.H., Senra A., Fernø J., Gómez-Valadés A.G., Schwaninger M. (2021). Sirt3 in POMC neurons controls energy balance in a sex- and diet-dependent manner. Redox Biol..

[21] Nóvoa E., da Silva Lima N., Gonzalez-Rellan M.J., Chantada-Vazquez M.D.P., Verheij J., Rodriguez A., Esquinas-Roman E.M., Fondevila M.F., Koning M., Fernandez U. (2025). Mitochondrial antiviral signaling protein enhances MASLD progression through the ERK/TNFα/NFκβ pathway. Hepatology.

[22] Gonzalez-Rellan M.J., Novoa E., da Silva Lima N., Rodriguez A., Veyrat-Durebex C., Seoane S., Porteiro B., Fondevila M.F., Fernandez U., Varela-Rey M. (2023). Hepatic p63 regulates glucose metabolism by repressing SIRT1. Gut.

[23] Pena-Leon V., Perez-Lois R., Villalon M., Folgueira C., Barja-Fernández S., Prida E., Baltar J., Santos F., Fernø J., García-Caballero T. (2024). Gastric GDF15 levels are regulated by age, sex, and nutritional status in rodents and humans. J. Endocrinol. Investig..

[24] Al-Massadi O., Quiñones M., Clasadonte J., Hernandez-Bautista R., Romero-Picó A., Folgueira C., Morgan D.A., Kalló I., Heras V., Senra A. (2019). MCH Regulates SIRT1/FoxO1 and Reduces POMC Neuronal Activity to Induce Hyperphagia, Adiposity, and Glucose Intolerance. Diabetes.

[25] Imbernon M., Sanchez-Rebordelo E., Romero-Picó A., Kalló I., Chee M.J., Porteiro B., Al-Massadi O., Contreras C., Fernø J., Senra A. (2016). Hypothalamic kappa opioid receptor mediates both diet-induced and melanin concentrating hormone-induced liver damage through inflammation and endoplasmic reticulum stress. Hepatology.

[26] Prida E., Pérez-Lois R., Jácome-Ferrer P., Muñoz-Moreno D., Brea-García B., Villalón M., Pena-Leon V., Vázquez-Cobela R., Aguilera C.M., Conde-Aranda J. (2024). The PTK2B gene is associated with obesity, adiposity, and leptin levels in children and adolescents. iScience.

[27] Porteiro B., Fondevila M.F., Delgado T.C., Iglesias C., Imbernon M., Iruzubieta P., Crespo J., Zabala-Letona A., Fernø J., González-Terán B. (2017). Hepatic p63 regulates steatosis via IKKβ/ER stress. Nat. Commun..

[28] Zhang X., Zhang G., Zhang H., Karin M., Bai H., Cai D. (2008). Hypothalamic IKKbeta/NF-kappaB and ER stress link overnutrition to energy imbalance and obesity. Cell.

[29] Nogueiras R., Habegger K.M., Chaudhary N., Finan B., Banks A.S., Dietrich M.O., Horvath T.L., Sinclair D.A., Pfluger P.T., Tschöop M.H. (2012). Sirtuin 1 and sirtuin 3: Physiological modulators of metabolism. Physiol. Rev..

[30] Contreras C., González-García I., Seoane-Collazo P., Martínez-Sánchez N., Liñares-Pose L., Rial-Pensado E., Fernø J., Tena-Sempere M., Casals N., Diéguez C. (2017). Reduction of Hypothalamic Endoplasmic Reticulum Stress Activates Browning of White Fat and Ameliorates Obesity. Diabetes.

[31] Malhi H., Kaufman R.J. (2011). Endoplasmic reticulum stress in liver disease. J. Hepatol..

[32] Ge X., Yang H., Bednarek M.A., Galon-Tilleman H., Chen P., Chen M., Lichtman J.S., Wang Y., Dalmas O., Yin Y. (2018). LEAP2 Is an Endogenous Antagonist of the Ghrelin Receptor. Cell Metab..

[33] Perelló M. (2025). Critical Insights Into LEAP2 Biology and Physiological Functions: Potential Roles Beyond Ghrelin Antagonism. Endocrinology.

[34] Lu X., Huang L., Huang Z., Feng D., Clark R.J., Chen C. (2021). LEAP-2: An Emerging Endogenous Ghrelin Receptor Antagonist in the Pathophysiology of Obesity. Front. Endocrinol..

[35] Cohen H.Y., Miller C., Bitterman K.J., Wall N.R., Hekking B., Kessler B., Howitz K.T., Gorospe M., de Cabo R., Sinclair D.A. (2004). Calorie restriction promotes mammalian cell survival by inducing the SIRT1 deacetylase. Science.

[36] Chalkiadaki A., Guarente L. (2012). Sirtuins mediate mammalian metabolic responses to nutrient availability. Nat. Rev. Endocrinol..

[37] Ramadori G., Lee C.E., Bookout A.L., Lee S., Williams K.W., Anderson J., Elmquist J.K., Coppari R. (2008). Brain SIRT1: Anatomical distribution and regulation by energy availability. J. Neurosci..

[38] Hou X., Xu S., Maitland-Toolan K.A., Sato K., Jiang B., Ido Y., Lan F., Walsh K., Wierzbicki M., Verbeuren T.J. (2008). SIRT1 regulates hepatocyte lipid metabolism through activating AMP-activated protein kinase. J. Biol. Chem..

[39] Purushotham A., Schug T.T., Xu Q., Surapureddi S., Guo X., Li X. (2009). Hepatocyte-specific deletion of SIRT1 alters fatty acid metabolism and results in hepatic steatosis and inflammation. Cell Metab..

[40] Rodgers J.T., Puigserver P. (2007). Fasting-dependent glucose and lipid metabolic response through hepatic sirtuin 1. Proc. Natl. Acad. Sci. USA.

[41] Wang R.H., Li C., Deng C.X. (2010). Liver steatosis and increased ChREBP expression in mice carrying a liver specific SIRT1 null mutation under a normal feeding condition. Int. J. Biol. Sci..

[42] Ozcan U., Cao Q., Yilmaz E., Lee A.H., Iwakoshi N.N., Ozdelen E., Tuncman G., Görgün C., Glimcher L.H., Hotamisligil G.S. (2004). Endoplasmic reticulum stress links obesity, insulin action, and type 2 diabetes. Science.

[43] Ozcan U., Yilmaz E., Ozcan L., Furuhashi M., Vaillancourt E., Smith R.O., Görgün C.Z., Hotamisligil G.S. (2006). Chemical chaperones reduce ER stress and restore glucose homeostasis in a mouse model of type 2 diabetes. Science.

