# Supplementary material for: Hepatic Olfr734 Deficiency Worsens Hepatic Glucose Metabolism and Induces MASLD in Mice
Source: Nutrients. 2025 Jul 25;17(15):2426. doi: 10.3390/nu17152426 (PMC12348634; doi:10.3390/nu17152426)
Supplement: Supplementary file 1 [file nutrients-17-02426-s001.zip › Table 1.docx]

| ID | Name | Sequence 5' -----3' |
| --- | --- | --- |
| 1 | β - actin Human Forward | CACAGAGCCTCGCCTTTGC |
| 2 | β - actin Human Reverse | CCACCATCACGCCCTGG |
| 3 | OLFR734 Forward | CTCGGGAAGTGCAGCTAGTC |
| 4 | OLFR734 Reverse | GCGGTCAAAAGCCATCACTG |
| 5 | FAS Forward | AGTGTTCGTTCCTCGGAGTG |
| 6 | FAS Reverse | GCTGCTGTTGGAAGTCAGC |
| 7 | CPT1a Forward | TTGTCGGAAGAAGAAAATGC |
| 8 | CPT1a Reverse | CAGCTGGCTGGTTGTTGTCA |
| 9 | LPL Reverse | GCTGAGTCCTTTCCCTTCTGCAG |
| 10 | LPL Forward | TCTGACATTTGCAGGTCTATCT |
| 11 | FGF21 Forward | AGATCAGGGAGGATGGAACA |
| 12 | FGF21 Reverse | TCAAAGTGAGGCGATCCATA |
| 13 | SREBP1 Forward | CGGGAAGTCACTGTCTTGGT |
| 14 | SREBP1 Reverse | GGTTTTGAACGACATCGAAGA |
| 15 | SREBP2 Reverse | ACCTAGACCTCGCCAAAGGT |
| 16 | SREBP2 Forward | GCACGGATAAGCAGGTTTGT |
| 17 | ACC Forward | TGGGCGGGATGGTCTCTTT |
| 18 | ACC Reverse | AGTCGCAGAAGCAGCCCATT |
| 19 | PGC1a Forward | AAGTGTGGAACTCTCTGGAACTG |
| 20 | PGC1a Reverse | GGGTTATCTTGGTTGGCTTTATG |
| 21 | INSING1 Forward | CACCCAGGACCAGTGTCTCT |
| 22 | INSING1 Reverse | TGGGAAACATAGGACGACAGT |
| 23 | INSING2 Forward | TCCACCACAGTCCTACTGACA |
| 24 | INSING2 Reverse | GGTATAAATCACGCCAGTGCT |
| 25 | IKKB Forward | TGCAGGACACTGTGAAGGAG |
| 26 | IKKB Reverse | CTGGCAGAGTGAGATGTCCA |
| 27 | AMPK1a Forward | GAATCTTCTGCCGGTTGAGT |
| 28 | AMPK1a Reverse | CCTTCGGGAAAGTGAAGGT |
| 29 | AMPK2a Forward | CAGTAATCCACGGCAGACAG |
| 30 | AMPK2a Reverse | CGACTACATCTGCAAACATGG |
| 31 | SIRT1 Forward | AGTTCCAGCCGTCTCTGTGT |
| 32 | SIRT1 Reverse | CTCCACGAACAGCTTCACAA |
| 33 | ATF4 Forward | GGGTTCTGTCTTCCACTCCA |
| 34 | ATF4 Reverse | AAGCAGCAGAGTCAGGCTTTC |
| 35 | CHOP Forward | CCACCACACCTGAAAGCAGAA |
| 36 | CHOP Reverse | AGGTGAAAGGCAGGGACTCA |
| 37 | XBP1un Forward | CAGCACTCAGACTATGTGCA |
| 38 | XBP1un Reverse | GTCCATGGGAAGATGTTCTGG |
| 39 | XBP1 Forward | CTGAGTCCGAATCAGGTGCAG |
| 40 | XBP1 Reverse | GTCCATGGGAAGATGTTCTGG |
| 41 | BIP Forward | TTCAGCCAATTATCAGCAAACTCT |
| 42 | BIP Reverse | TTTTCTGATGTATCCTCTTCACCAGT |
| 43 | OR4M1 Forward | GGATAAAGTCCTCCACTGTGGT |
| 44 | OR4M1 Reverse | AGGCCATCACTGTGAGCAAG |
