# Supplementary figures and images for: Hepatic Olfr734 Deficiency Worsens Hepatic Glucose Metabolism and Induces MASLD in Mice
Source: Nutrients. 2025 Jul 25;17(15):2426. doi: 10.3390/nu17152426 (PMC12348634; doi:10.3390/nu17152426)

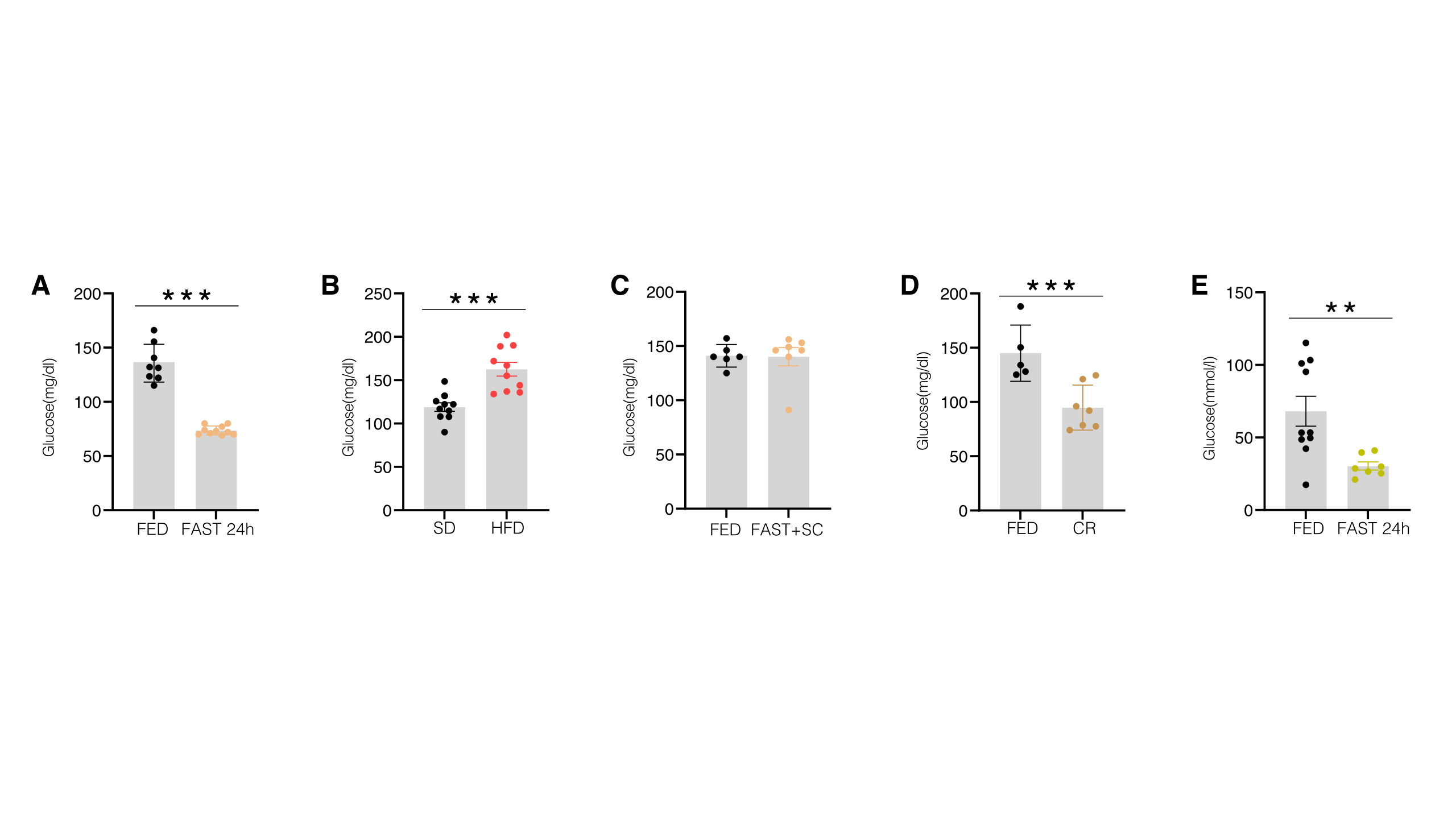

Supplement: Supplementary file 1 [file nutrients-17-02426-s001.zip › FS1 (5).png]

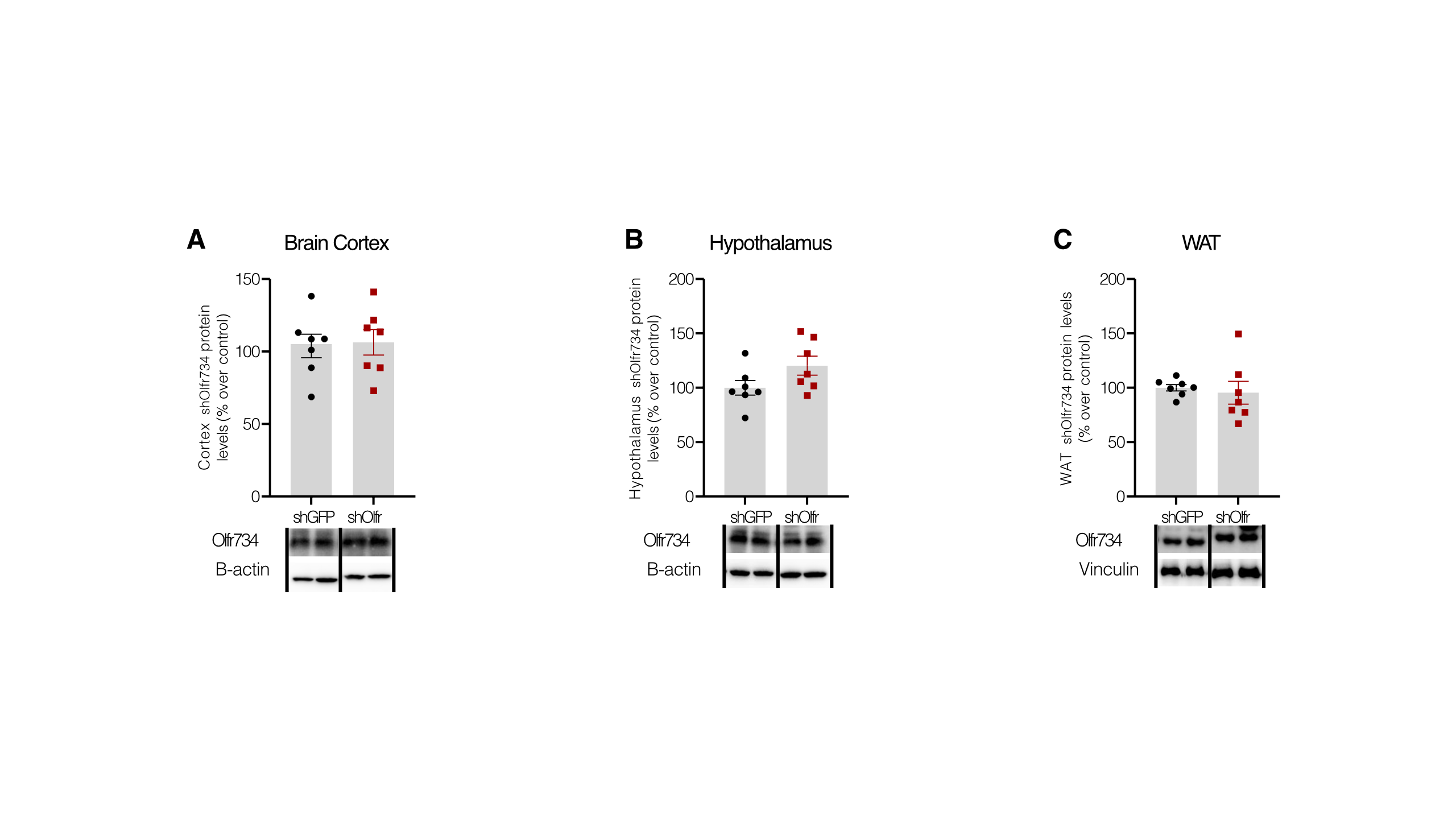

Supplement: Supplementary file 1 [file nutrients-17-02426-s001.zip › FS2 (5).png]

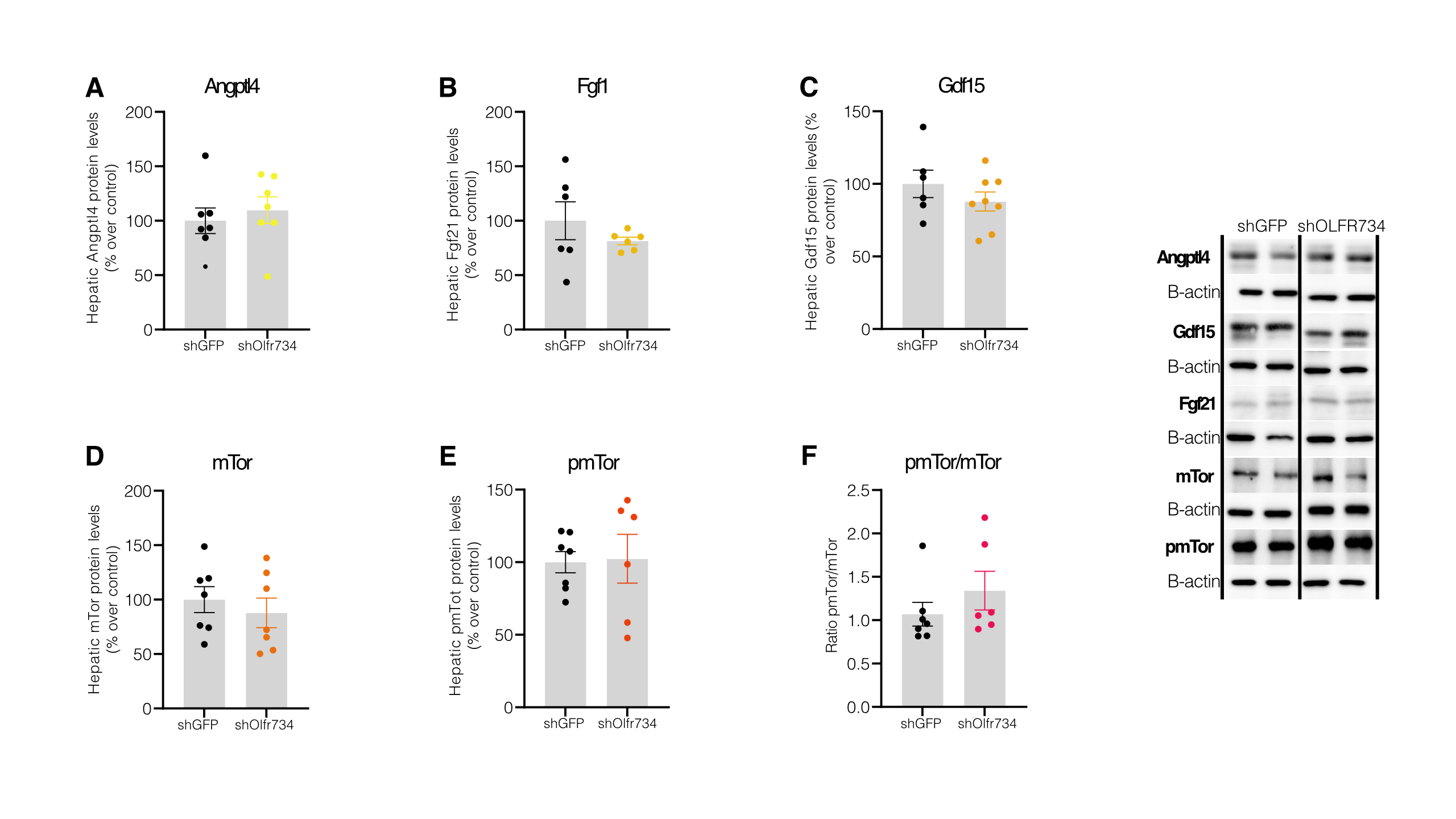

Supplement: Supplementary file 1 [file nutrients-17-02426-s001.zip › FS3 (2).png]
